# Strengthened social ties in disasters: Threat-awe encourages interdependent worldviews via powerlessness

**DOI:** 10.1371/journal.pone.0285049

**Published:** 2023-04-26

**Authors:** Ryota Takano, Michio Nomura

**Affiliations:** 1 Department of Social Psychology, The University of Tokyo, Tokyo, Japan; 2 Japan Society for the Promotion of Science, Tokyo, Japan; 3 Graduate School of Education, Kyoto University, Kyoto, Japan; Walden University, UNITED STATES

## Abstract

Threat-awe, a negatively valenced variant of awe, is thought to strengthen social ties among community members. However, few empirical studies have examined the social functions of threat-awe. This study investigated whether threat-awe is linked to interdependent worldviews through feelings of powerlessness in comparison with positive awe. After remembering and describing their experiences of positive-or threat-awe, 486 Japanese participants reported on items regarding a small self, a sense of powerlessness, and interdependent worldviews. The results demonstrated that threat-awe encouraged interdependent worldviews via an increased sense of powerlessness, rather than the small self, compared to the positive awe condition. From textual perspectives, the semantic networks between awe-related and other words differed from the descriptions of threat-awe and positive awe experiences. These results provide a more nuanced understanding of the emotions of awe as well as new insights into human cooperation in the context of disasters.

## Introduction

Understanding how individuals respond to significant events that are beyond human knowledge, such as natural or human-made disasters, is a critical area of inquiry within the social sciences, including psychology and linguistics. The impact of such events on people’s narratives, cognitions, and social behaviors can be overreaching, potentially altering the dynamics of cooperation and coordination within human societies [1, 2]. In psychology, awe is identified as one of the representative emotions that arise in such situations, which is defined as an emotional response to stimuli that are conceptually and perceptually vast and transcend current frames of reference [3]. Awe is thought to be more than a feeling that changes the sense of self (e.g., a small self), thereby impacting beliefs, mental health, and social behavior [4, 5]. Importantly, awe can be tinged with both positive and negative valence depending on the characteristics of the experience. Prior research has identified threat-awe, which is triggered by threatening stimuli, as having different functions than positive awe [6–8]. Crucially, some studies have suggested that threat-awe elicited by experiencing natural disasters strengthens social ties among community members for group survival [9, 10]. However, few empirical studies have investigated the specific psychological and linguistic mechanisms underlying the social function of threat-awe. Given that threat-awe is related to an increased sense of powerlessness, the present study examined whether threat-awe is associated with interdependent worldviews through feelings of powerlessness in comparison with positive awe, while focusing on how people narrate the experiences of awe.

Keltner and Haidt (2003) identified two central features of awe using a prototype approach: the need for accommodation, which is the process by which a person revises or creates a new mental schema to account for the discrepancy between the stimuli and their current understanding of the world; and the perception of vastness, which is the sense that an individual has encountered something immense [3]. Consistent with this framework, previous studies have demonstrated that changes in the sense of self is a key characteristic of awe, which predicts various behavioral outputs induced by it. For example, experimentally induced awe increases one’s perception of their smallness, which in turn promotes prosocial behavior, humility, and group engagement [5, 11, 12].

Importantly, threat is one of the features that characterize awe experiences [3]. Previous studies have demonstrated how the threat-awe that arises in response to threatening awe stimuli (e.g., tornadoes, wrathful gods) is associated with different psychological, physiological, and neural responses than positive forms of awe, which arise during aesthetic experiences (e.g., beautiful nature, spiritual phenomena, and the virtue of a charismatic leader) [6, 8, 13]. For instance, threat-awe is associated with lower levels of subjective well-being and activation of the sympathetic nervous system [6]. Notably, the altered sense of self during the threat-awe experience is called “powerlessness,” which indicates in individuals a feeling that they have little power over the awe-related situations, and is therefore associated with decreased subjective well-being [6, 7].

Regarding the social function of threat-awe, some studies suggest that the experiences of threat-awe, such as natural disasters, encourage interdependent worldviews through a smaller sense of self, therefore strengthening social ties among community members [9, 10]. Furthermore, a cross-sectional study showed that the feelings of awe induced by watching a threat-awe video were positively related to the willingness to help people in need via a smaller sense of self and a sense of global community [10]. The next step is to investigate the specific mechanisms underlying the promoting effects of threat-awe experiences on interdependent worldviews by comparing positive forms of awe. The sense of powerlessness might explain the relationship between threat-awe and interdependent worldviews as it may encourage cooperation among community members to overcome strenuous situations.

Previous studies have demonstrated that the textual context of an emotional experience can have a significant impact on subsequent thoughts, attitudes, and behavior; therefore, it is important to address how emotional experiences are expressed in natural language [14, 15]. Specifically, analyzing the narrative of an emotional experience is a valuable tool for a deeper understanding of complex emotions such as awe. It should be noted that there are two main Japanese words for awe, ‘*ikei* (畏敬)’ and ‘*ifu* (畏怖),’ which have different ideography [15]. While *ikei* conveys respect or a positive evaluation, as indicated by the second character ‘敬,’ *ifu* involves negative feelings since the second character ‘怖’ means being afraid. Consistent with this, previous empirical research in Japan revealed that the ratings of *ifu* were higher in the threat-awe condition, while those of *ikei* were higher in the positive awe condition [8]. Furthermore, research has demonstrated that both the experiences of positive awe and threat-awe can elicit feelings of awe; however, the specific contexts in which these experiences occur may differ [6, 8, 13]. Based on these observations, threat-awe experiences would be more frequently labeled as *ifu* than as *ikei*, thereby being linked to specific semantic networks between these awe-related words and others (e.g., those that have social meanings such as family). Using text mining to describe awe experiences, this study explored whether threat-awe experiences had different co-occurrence networks between awe-related words and other words than positive awe experiences did.

It is important to examine these psychological and linguistic processes in the cultural and historical context of Japan because natural disasters, which are typical examples of threat-awe experiences, occur frequently in the country due to its climate and topography [16]. The Great Hanshin-Awaji Earthquake and the Great East Japan Earthquake were the two major natural disasters in Japan that occurred in the last few decades. The Great Hanshin-Awaji Earthquake occurred on January 17, 1995, in the southern part of Hyogo Prefecture, with a magnitude of 7.3 and led to more than 4,000 casualties. The Great East Japan Earthquake, which occurred on March 11, 2011, had its epicenter in the Pacific Ocean, east of the Oshika Peninsula of the Tohoku region, with a magnitude of 9.0 and led to more than 20,000 casualties. It should be noted that the experiences of threat-awe, such as these earthquakes, have a significant impact on people in that culture, including those not directly affected by the disaster, through social media channels such as news and social networking services. In this study, we also focused on surveying people in Japan about their narratives of threat-awe experiences, considering the country’s cultural and historical backgrounds. Japanese participants are generally thought to harbor a reverence for nature due to the role of the Shinto religion in Japanese culture [17], and it is thought that this led many Japanese participants to recall several awe experiences related to nature.

In summary, the present study aimed to investigate whether the experiences of threat-awe encourage interdependent worldviews via feelings of powerlessness through a comparison with those of positive awe among people in Japan. We tested the following hypotheses:

**H1**. Threat-awe experiences are associated with higher levels of feelings of powerlessness and interdependent worldviews than positive awe experiences.
**H2**. The promoting effects of threat-awe on interdependent worldviews are mediated by increased feelings of powerlessness.
**H3**. Threat-awe experiences are more frequently labeled as *ifu* than *ikei*.
**H4**. The co-occurrence networks of the descriptions of threat-awe experiences between awe-related (i.e., *ikei* and *ifu*) and other words are different from those of positive awe experiences.

## Methods

### Participants

This study recruited 644 Japanese participants through a Japanese crowdsourcing service, Crowd Works (Table 1). Due to incorrect answers to the attention check question, 158 participants were excluded, leaving a final *N* = 486 (190 males, 295 females, and one other, *M*_*age*_ = 38.83 years, *SD* = 10.77, *Range*: 20–73). The target sample size was determined using a priori power analysis (G*Power) [18], which indicated that the required sample size was 414 to detect the effects of interest (*d* = .32, the average effect size from [6], Study 2b) in an independent *t*-test, with α = 0.05 and statistical power = 0.90. The study conformed to the principles expressed in the Declaration of Helsinki and was approved by the local ethics committee at Graduate School of Education, Kyoto University (Ref-No. CPE-198) and all participants provided written informed consent before their participation. This study was not preregistered. The data and analysis codes are available at: https://osf.io/x4ujt/?view_only=a52d767556f14c8eb289f8406fe4e6f0.

**Table 1:** Demographic characteristics of participants.

Demographic Characteristics	Classification	Sample Size or Mean	
		Positive Awe Condition (*N* = 242)	Threat-awe Condition (*N* = 244)
Age		38.17 years	39.49 years
Gender	Men	93	97
	Women	148	147
	Other	1	0

### Procedures and materials

Participants were randomly assigned to one of two conditions: a positive awe condition (*N* = 242, 93 males, 148 females, and one other, *M*_*age*_ = 38.17 years, *SD* = 10.01, *Range*: 20–69) or a threat-awe condition (*N* = 244, 97 males and 147 females, *M*_*age*_ = 39.49 years, *SD* = 11.47, *Range*: 20–73). There were no statistically significant differences in the mean age and sex distributions between the two awe conditions (age: *b* = 1.31, 95% CI [− 0.60, 3.23], *p* = .178, gender: χ^2^ = 0.09, *p* = .765). Participants were asked to recall and describe their experiences of awe (modified from [6], Study 2b). They presented an example of awe experiences with a picture of the aurora (positive awe condition) or the tsunami (threat-awe condition), which were validated by a separate pilot study (see S1 File). Participants were instructed to “Please take a moment to recall a time when you felt intense *ikei* or *ifu* like the examples described above or depicted in the picture below.”

After the recall task, they reported how intensely they felt 13 emotions, such as *ikei* and *ifu*, using 9-point scales (1 = *not at all*, 9 = *extremely*) [8], and chose either *ikei* or *ifu* as the most representative emotion during the experience (*ikei*-*ifu* selections). Participants were then asked to select an elicitor (i.e., what triggered the experience) for the experience from 10 candidates (a spiritual/religious experience, another person, architecture, art, idea, music, nature, natural disasters, the self, and other; [11], the options of nature and natural disasters were then combined into one as “nature”), followed by indicating the degree to which they experienced a smaller sense of self [11, 19], a sense of powerlessness (α = .65, [6]), and a feeling of threat on a manipulation check (7-point scale, 1 = *not at all*, 7 = *extremely*). These questionnaires of emotions and sense of self were frequently used in the studies of awe [5, 6, 8, 19]. To assess the interdependent worldviews, participants rated their agreement with three statements on a 7-point scale (1 = *not at all*, 7 = *extremely*; α = .89): “How did you feel like to care for your family and friends?” “How did you feel like to cherish the regional ties?” and “How important do you think it is to help each other?” (the authors translated original Japanese words into English). The items were borrowed from a public survey conducted by the Japanese Cabinet Office in 2012, which is often used in public opinion polls and academic surveys [20, 21]. Furthermore, for exploratory purposes, participants also rated their agreement with two statements on a 7-point scale (1 = *not at all*, 7 = *strongly*) to assess the perception of vastness and need for accommodation, which were thought to be awe-related psychological processes (modified from [6, 22]): “I perceived a sense of vastness” and “My worldviews changed,” respectively. All questions began with the sentence, “When you experienced *ikei* (*ifu*)…” depending on the choice of *ikei*-*ifu* selection. S1 Table in S1 File includes all the items of these measures. Correlations between items within the interdependent worldviews and the sense of powerlessness scales were listed in S2 and S3 Tables in S1 File. Participants were also asked to answer items from other questionnaires (e.g., perceived distance between oneself and the elicitor), which will serve other, related investigations. All items are available at: https://osf.io/x4ujt/?view_only=a52d767556f14c8eb289f8406fe4e6f0. Items originally created in English were translated into Japanese by two authors based on the advice of one bilingual researcher.

### Statistical analyses

**Fig 1:**
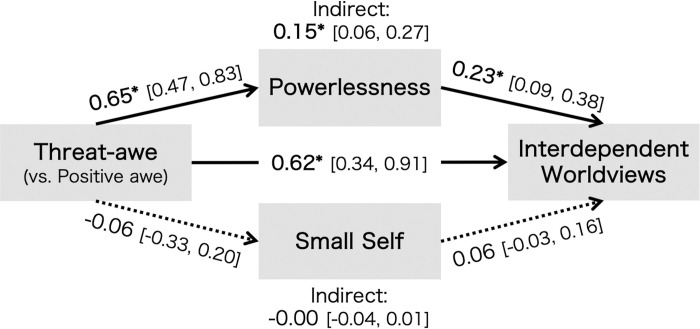
Mediation analysis of awe condition on interdependent worldviews via powerlessness and small self. *Note*. Values indicate standardized regression coefficients. The dotted line indicates the insignificant path coefficient. *p < .05.

Data analyses were conducted using the R software (v. 4.2.0). Linear regression analyses were performed using a robust estimation method to investigate the differences in mean scores of variables between the positive and threat-awe conditions using the “lm_robust” function in the estimatr package [23]. The two awe conditions were represented using dummy coding (positive awe = 0, threat-awe = 1). Correlation analyses for each condition were performed to examine the relationships between the variables (S4 and S5 Tables in S1 File). The lavaan package [24] was used, along with maximum likelihood (ML) estimation, to specify a structural equation model (SEM) in which the interdependent worldview on awe conditions was regressed, mediated by feelings of powerlessness or a small self (see Fig 1). To test whether powerlessness or the small self explains the effect of awe manipulation on interdependent worldviews, mediation analyses were performed with bias-corrected 95% CIs (5,000 bootstrap resamples). We reported confidence intervals rather than *p*-values for testing indirect effects using bias-corrected bootstrap methods according to previous studies [25–27]; therefore, the coefficient was deemed statistically significant when 95% confidence intervals did not include 0 for indirect effects.

**Fig 2:**
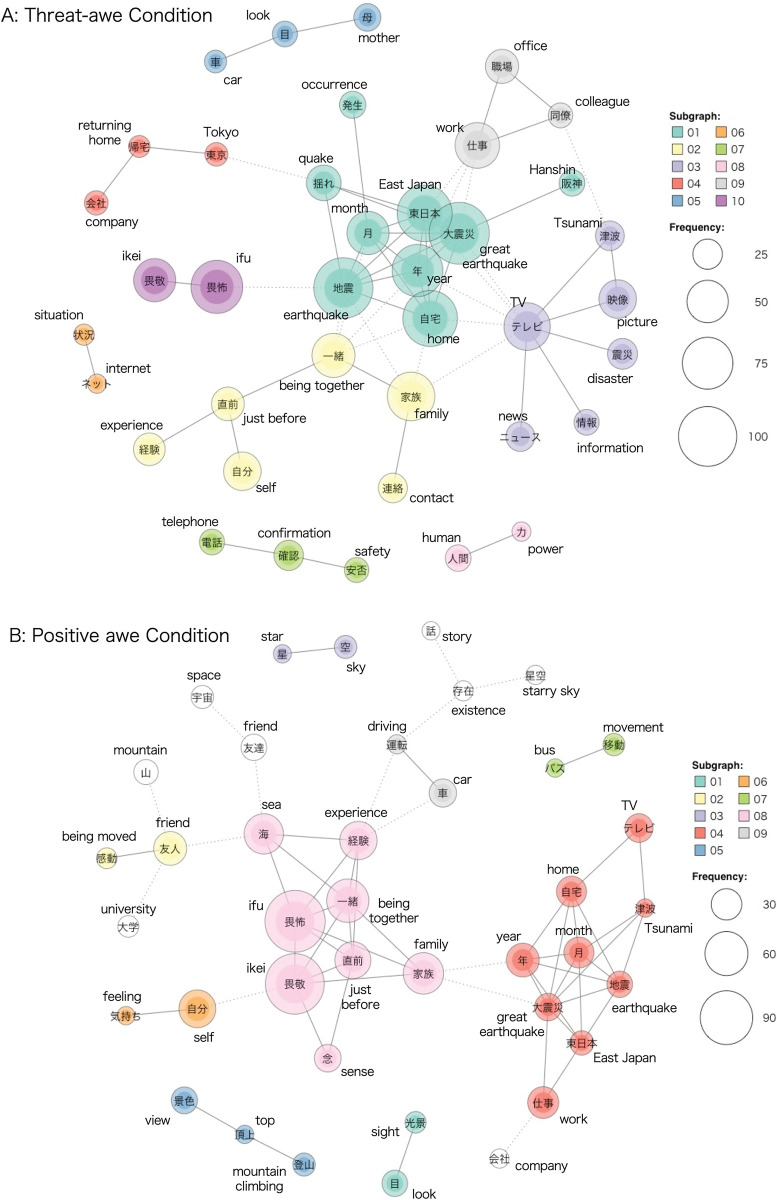
Co-occurrence network of words in descriptions about (A) threat-awe and (B) positive awe experiences. *Notes*. The minimum frequency of each word was set at 10, and the number of edges was set at 60. The lines indicate a strong co-occurrence relationship between the words. The size of the circle indicates the frequency, and its color indicates a subgraph. The dotted line indicates the link with a word in the other sub-graphs. The authors translated Japanese words into English.

Furthermore, this study examined whether the choice proportions of *ikei*-*ifu* selections differed between the positive and threat-awe conditions by performing logistic regression analyses with the robust estimation method. Standard errors were estimated by using the “standard_error_robust” function in the parameters package. Regarding the text analyses, the distributions of the word frequency and their co-occurrence networks in the descriptions of both awe experiences were identified with the KH coder (ver. for mac 3.Beta.05b, https://khcoder.net/en/), which is one of the most used text mining software for Japanese text analyses [28, 29]. First, nouns from the descriptions of each condition were extracted and their frequencies were computed. Second, a word co-occurrence network analysis was performed to investigate the semantic network of each awe experience, with a particular focus on “*ikei*” and “*ifu*.” In this analysis, words that had an occurrence pattern were connected to one another based on the Jaccard coefficient, which was calculated as the number of sentences containing two words, divided by the number of sentences containing them. Larger circles indicate words used more frequently (see Fig 2). This analysis automatically detects and groups subgraphs that contain relatively strongly connected words. The minimum frequency of a word was 10, and the number of edges was set to 60, which is suitable for visually analyzing a network [30].

## Results

Regarding the elicitor of the experience, there were significant differences in the distributions of elicitors (threat-awe: *N*_*religious experience*_ = 8, *N*_*another person*_ = 36, *N*_*architecture*_ = 2, *N*_*art*_ = 1, *N*_*idea*_ = 6, *N*_*music*_ = 2, *N*_*nature*_ = 184, *N*_*the self*_ = 0, *N*_*other*_ = 5; positive awe: *N*_*religious experience*_ = 18, *N*_*another person*_ = 22, *N*_*architecture*_ = 12, *N*_*art*_ = 15, *N*_*idea*_ = 7, *N*_*music*_ = 4, *N*_*nature*_ = 159, *N*_*the self*_ = 2, *N*_*other*_ = 3). Positive awe was elicited significantly more frequently by spiritual/religious experiences, art, and architecture than threat-awe (*χ*^*2*^s > 4.15, *p*s < .041). However, threat-awe was elicited more frequently by nature (including natural disasters) than positive awe (*χ*^*2*^ = 5.51, *p* = .019), which is consistent with previous studies [6]. Note that a small number of observations, except for nature, might invalidate inferences from these investigations.

Regarding the differences in manipulation checks and emotional responses between conditions, the linear regression models with the robust estimation method revealed that the ratings of threat and *ifu* were statistically significantly higher in the threat-awe condition than in the positive awe condition (threat: *M*_*Threat-awe*_ = 6.25, *M*_*Positive awe*_ = 4.93, *b* = 1.32, 95% CI [1.04, 1.60], *p* < .001; *ifu*: *M*_*Threat-awe*_ = 7.66, *M*_*Positive awe*_ = 6.74, *b* = 0.92, 95% CI [0.59, 1.26], *p* < .001). Meanwhile, participants in the positive awe condition reported higher levels of *ikei* than those in the threat-awe condition (*M*_*Threat-awe*_ = 5.50, *M*_*Positive awe*_ = 6.81, *b* = –1.31, 95% CI [–1.74, –0.88], *p* < .001). In addition, the ratings of accommodation were equally high in both awe conditions (*M*_*Threat-awe*_ = 4.88, *M*_*Positive awe*_ = 4.94, *b* = −0.06, 95% CI [−0.34, 0.22], *p* = .692). Meanwhile, participants in the threat-awe condition reported a lower level of perceived vastness than those in the positive awe condition (*M*_*Threat-awe*_ = 3.52, *M*_*Positive awe*_ = 4.98, *b* = − 1.46, 95% CI [−1.79, −1.13], *p* < .001), revealing consistency with previous studies [6, 8] (please note that these analyses were conducted exploratorily). In line with H1, participants in the threat-awe condition reported a higher sense of powerlessness and interdependent worldviews than those in the positive awe condition (powerlessness: *M*_*Threat-awe*_ = 5.72, *M*_*Positive awe*_ = 5.07, *b* = 0.65, 95% CI [0.47, 0.83], *p* < .001; interdependent worldviews: *M*_*Threat-awe*_ = 5.17, *M*_*Positive awe*_ = 4.40, *b* = 0.77, 95% CI [0.50, 1.04], *p* < .001). The effects of threat-awe on the sense of powerlessness and interdependent worldviews remained even after controlling the effects of fear ratings, age, gender (powerlessness: *b* = 0.24, 95% CI [0.04, 0.44], *p* = .016; interdependent worldviews: *b* = 0.58, 95% CI [0.27, 0.89], *p* < .001). There was no statistically significant difference in the ratings of the small self between conditions (*M*_*Threat-awe*_ = 1.97, *M*_*Positive awe*_ = 2.03, *b* = − 0.06, 95% CI [− 0.33, 0.20], *p* = .649). Other differences in the ratings of emotional responses also emerged (S6 Table in S1 File).

A mediation analysis was performed to test whether a sense of powerlessness mediates the relationship between awe conditions and interdependent worldviews (Fig 1). The results showed a statistically significant indirect effect of the threat-awe condition on interdependent worldviews via a sense of powerlessness (*b* = 0.15, 95% CI [0.06, 0.27]), and the direct effect remained (*b* = 0.62, 95% CI [0.34, 0.91]). Meanwhile, there was no statistically significant indirect effect of awe conditions on interdependent worldviews through the sense of a small self (*b* = –0.00, 95% CI [–0.04, 0.01]). Therefore, in line with H2, the effect of threat-awe on interdependent worldviews was partially mediated by increased feelings of powerlessness and not the sense of a small self.

Furthermore, consistent with H3, there were statistically significant differences in the distributions of *ikei*-*ifu* selection (0 = *ikei*, 1 = *ifu*) between conditions (threat-awe: *N*_*ikei*_ = 20, *N*_*ifu*_ = 224; positive awe: *N*_*ikei*_ = 138, *N*_*ifu*_ = 104, *χ*^*2*^ = 132.02, *p* < .001, *b* = 2.70, *SE* = 0.27, *p* < .001), indicating that while threat-awe experiences are mainly labeled as *ifu*, positive awe experiences are labeled by *ikei* and *ifu* equally. In line with these results, as shown in Fig 2A, co-occurrence network analyses showed that *ifu* was used more frequently than *ikei* in the threat-awe condition. In this network, earthquake-related words had central roles, which were associated with awe-related subgraphs (*ikei* and *ifu*, subgraph No. 10 in Fig 2A), society-related subgraphs including family and colleagues (subgraph No. 02 in Fig 2A), and news-related subgraphs including TV and pictures (subgraph No. 03 in Fig 2A). These words mainly indicated two earthquakes in Japan: the Great Hanshin Awaji Earthquake in 1995 and the Great East Japan Earthquake in 2011. Meanwhile, as shown in Fig 2B, *ikei* and *ifu* were equally used in the positive awe condition, which had central roles in the network and were related to other subgraphs that included various words (e.g., sea, family, friend, car, self, and disaster). Therefore, the semantic network between *ikei* or *ifu* and other words is different between the threat-awe and positive awe conditions, which is consistent with H4.

## Discussion

This is the first study to investigate the psychological mechanisms underlying the effects of threat-awe experiences on social ties. Specifically, whether threat-awe increases interdependent worldviews via a sense of powerlessness compared to positive awe, was examined. To gain a more precise understanding of these processes in a textual and cultural context, this study also investigated whether the frequencies of *ikei* or *ifu* (two words meaning awe in Japanese) and their semantic network with other words differed between the descriptions of threat-awe and positive awe experiences. Consistent with the hypotheses, the results showed that the promoting effect of threat-awe on interdependent worldviews was mediated by an increased sense of powerlessness, while controlling for the small self. Furthermore, threat-awe experiences are more frequently labeled as *ifu*, whereas positive awe experiences are equally labeled as *ikei* and *ifu*. The co-occurrence network analyses showed that the words relating to earthquakes played central roles and were linked to awe-related, society-related, and news-related subgraphs in the threat-awe condition, while *ikei* and *ifu* played central roles, which were related to various other words in the positive awe condition.

In line with previous studies [6, 9, 10], threat-awe experiences increased the sense of powerlessness and interdependent worldviews. By comparing with positive awe, this study first demonstrated that a sense of powerlessness significantly mediated the relationship between threat-awe and increased interdependent worldviews. The sense of powerlessness is a feeling that individuals have little power during the threat-awe experiences [6]. These findings expand the existing knowledge regarding the psychological process of threat-awe, suggesting that the sense of powerlessness might play an important role in the function of threat-awe, that is, strengthening social ties among community members under harsh situations such as natural disasters.

As textual processes related to how powerlessness worked on interdependent worldviews, threat-awe experiences were more labeled as *ifu*, which is consistent with its ideographical feature that the second character “怖” means “being afraid.” Moreover, co-occurrence network analyses showed that earthquake-related words were central to the network and were associated with awe-related, society-related, and news-related words. As shown in Fig 2A, the earthquake-related words indicate two great earthquakes in the past couple of decades in Japan (the Great Hanshin Awaji and the Great East Japan Earthquake). These findings suggest that emotional words, such as *ikei* and especially *ifu*, are linked to society-related words, including family and friends, through the recollection of one’s own earthquake experiences.

It should also be noted that the positive awe manipulation in this study involved equal labels for *ikei* and *ifu*, as well as various kinds of elicitors and other subgraphs in the co-occurrence network. From the theoretical perspective, the threat-awe is thought to be a “primordial” emotional reaction to high-status individuals or agencies that has evolved over time [3, 6]. As time has passed, the scope of awe has been extended to include objects with similar qualities to high-status agencies, resulting in more generalized and widely prevalent forms of positive awe. Thus, our findings suggest that the recall manipulation used to induce positive awe in this study may activate a wide range of awe-inspiring events, which is consistent with this theoretical perspective.

Overall, our findings have theoretical and practical implications for the entire field of social sciences, particularly social psychology, disaster science, disaster management, and policymaking. The promoting effect of threat-awe on interdependent worldviews suggested that the powerlessness felt by an individual could rather drive forces to overcome the desperate situation by strengthening group cohesion, which was related to linguistic networks centered on earthquake-related words. Thus, we revealed psychological and linguistic mechanisms underlying how people cooperated under significant threatening situations, by focusing on the social function of awe, thereby contributing to the understanding of the basic principles of human social behavior. Furthermore, given that participants in this study were not limited to the actual victims of the disaster, these insights may inform the development of effective strategies for disaster management and policymaking that consider the psychological and emotional factors that influence human behavior in disaster situations. They do so by involving not only the victims, but also those who were indirectly affected by the disaster.

This study has several limitations, which may also be considered as scope for further research. First, the demographic characteristics not examined in this study might affect the effect of threat-awe. Although the effects of age and gender had no effects, other variables such as the geography, residences, and disaster experience may play a role. In particular, given that participants in this study were not limited to the actual victims of the disaster, the view of whether people live in regions that suffered “actual” harm from the disaster would be important for future research [22]. Second, in this study, the target of cooperation was limited to familiar people, such as friends and family (i.e., in-group members). Given that in-group favoritism could lead individuals to derogate out-groups [31], it would be interesting to examine whether threat-awe can increase negative attitudes toward out-group members. Third, although a sense of powerlessness significantly mediated the relationships between threat-awe and interdependent worldviews, the mediation effect was partial. Therefore, it is important to explore other possible mechanisms underlying the social functions of threat-awe such as nature connectedness, which has positive indirect effects between threat-awe and well-being [7]. Fourth, although this study focused only on Japanese samples, which allowed us to examine this subject based on its cultural and historical background, comparisons with other cultures would provide a more comprehensive understanding of the function of threat-awe [9, 32].

In summary, the present study indicates that threat-awe strengthens interdependent worldviews via an increased sense of powerlessness. Furthermore, this study identified specific semantic networks between *ikei* or *ifu*, the two words for awe in Japanese, and other words in the descriptions of threat-awe experiences. The current results provide a more nuanced understanding of the concept of awe, as well as new insights into the psychological mechanisms underlying human cooperation or coordination in the context of disasters.

## Supporting information

S1 File Supporting information file. (DOCX)
