# Supplementary material for: Strengthened social ties in disasters: Threat-awe encourages interdependent worldviews via powerlessness
Source: PLoS One. 2023 Apr 26;18(4):e0285049. doi: 10.1371/journal.pone.0285049 (PMC10132671; doi:10.1371/journal.pone.0285049)
Supplement: S1 File — (DOCX) [file pone.0285049.s001.docx]

# Supporting Information File

### Details of the Recall Task

Parcitipants were asked to recall and describe their experiences of threat-awe or positive awe [1]. In the threat-awe condition, they were presented the example of awe experiences with a picture of the tsunami as follow:


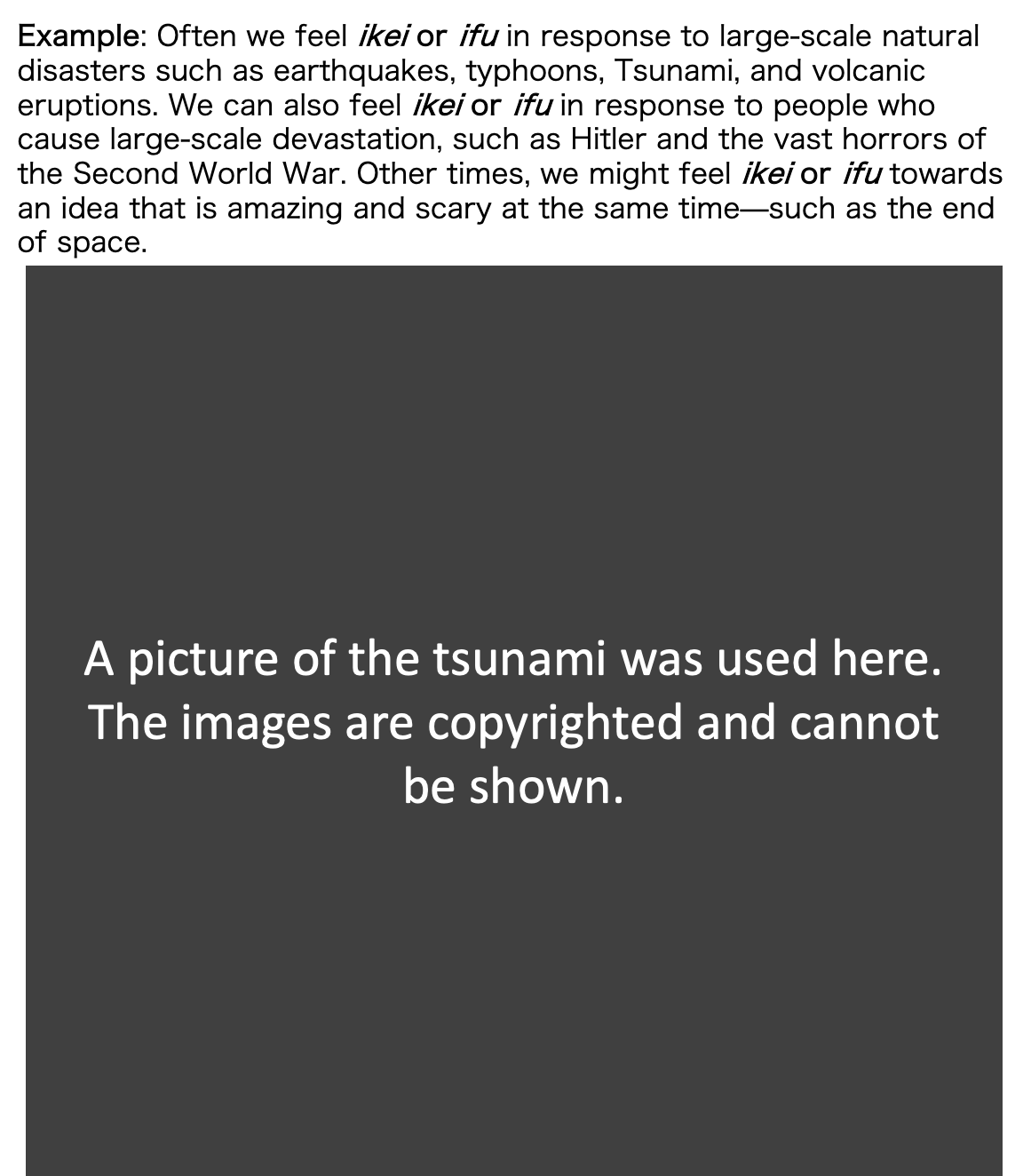


In the positive awe condition, they were presented the example of awe experiences with a picture of the aurora as follow:


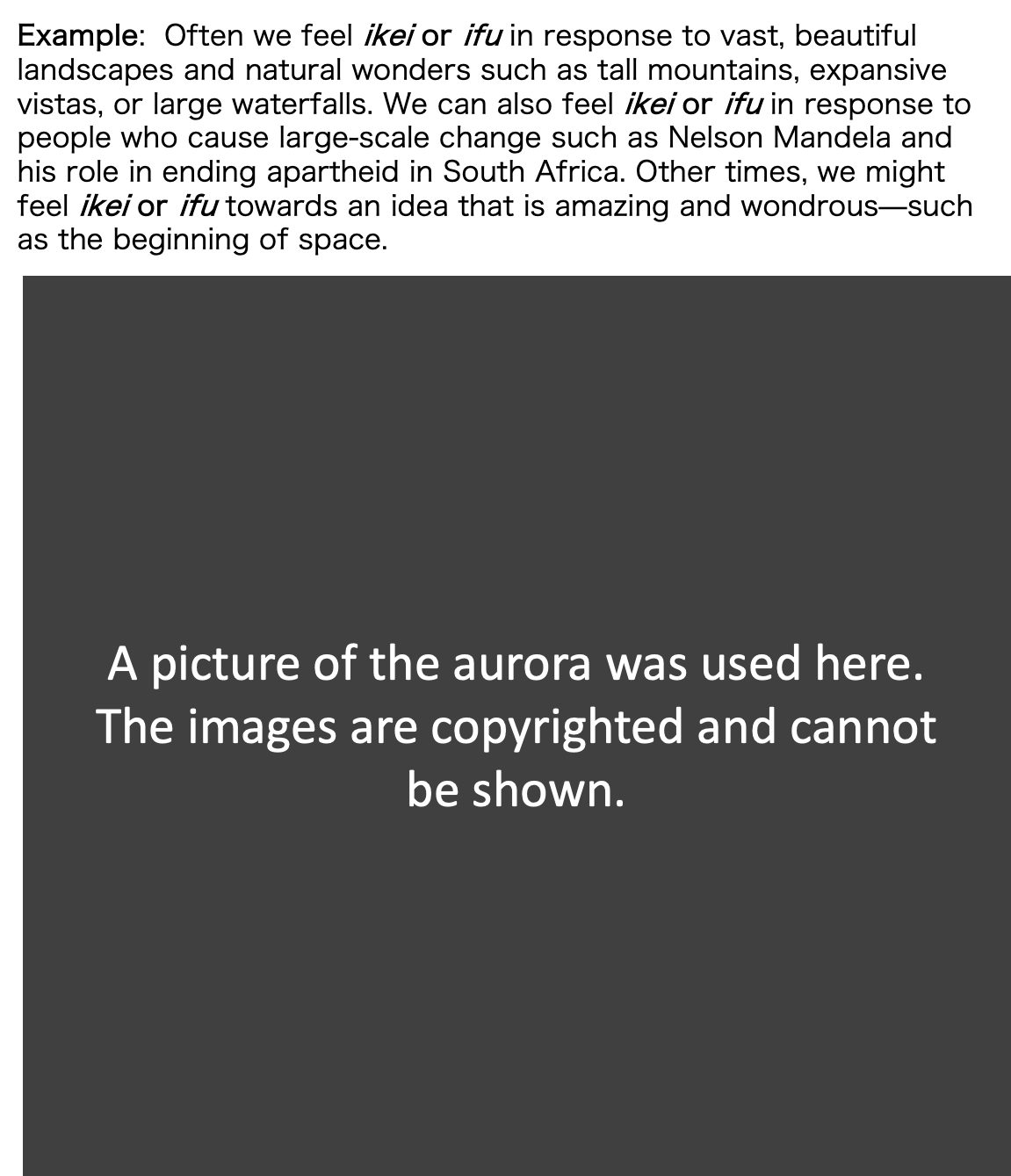


Participants were instructed, “please take a moment to recall a time when you felt intense ikei or ifu like the examples described above or depicted in the picture below.”

### Self-Reported Validation of Picture Stimuli

A pilot survey was conducted to validate the expected results. Seventeen participants looked at each picture in a randomized order (9 males and 8 females, *M_age_* = 21.47 ± 8.02). They reported how intensely they felt 16 emotions, including *ikei*, *ifu*, and fear using 9-point scales (1 = *not at all*, 9 = *extremely*). The mean *ifu* and fear scores were higher for videos of threat-awe condition than for videos of positive awe condition, while the mean ikei socres were higher in the positive awe condition than those in the threat-awe condition (*ifu*: *M_Threat-awe_* = 7.41; *M_Positie awe_* = 5.06, *d* = 1.29, 95% CI [0.56, 2.02], *p* < .001; fear: *M_Threat-awe_* = 8.24; *M_Positie awe_* = 3.24, *d* = 2.76, 95% CI [1.82, 3.70], *p* < .001; *ikei*: *M_Threat-awe_* = 5.24; *M_Positie awe_* = 7.12, *d* = –0.95, 95% CI [–1.64, –0.25], *p* = .001).

S1 Table.

*Items of the sense of powerlessness, the perception of threat, and interdependent worldviews*

| Variables | Items | Condition | *Mean* | *SD* |
| --- | --- | --- | --- | --- |
| Powerlessness | 1. When you experienced *ikei* (*ifu*), how much did you feel powerless? | Threat-awe | 6.08 | 1.24 |
|  |  | Positive awe | 5.19 | 1.74 |
|  | 2. When you experienced *ikei* (*ifu*), how much did you feel like you had control over your life? (reversed item) | Threat-awe | 5.17 | 1.59 |
|  |  | Positive awe | 4.87 | 1.53 |
|  | 3. When you experienced *ikei* (*ifu*), how much did you feel that what happened in my life was beyond my control? | Threat-awe | 6.85 | 1.10 |
|  |  | Positive awe | 6.19 | 1.27 |
|  | 4. When you experienced *ikei* (*ifu*), how much did you feel like that you had little control over the things that happened to you? | Threat-awe | 5.80 | 1.43 |
|  |  | Positive awe | 5.20 | 1.56 |
| Threat | When you experienced *ikei* (*ifu*), how much did you feel threatened? | Threat-awe | 6.25 | 1.15 |
|  |  | Positive awe | 4.93 | 1.93 |
| Interdependent Worldviews | 1. When you experienced *ikei* (*ifu*), how did you feel like to care for your family and friends? | Threat-awe | 5.56 | 1.65 |
|  |  | Positive awe | 4.91 | 1.64 |
|  | 2. When you experienced *ikei* (*ifu*), how did you feel like to cherish the regional ties? | Threat-awe | 4.75 | 1.78 |
|  |  | Positive awe | 3.94 | 1.76 |
|  | 3. When you experienced *ikei* (*ifu*), how important do you think is helping each other? | Threat-awe | 5.22 | 1.66 |
|  |  | Positive awe | 4.36 | 1.75 |
| Perception of Vastness | I perceived a sense of vastness. | Threat-awe | 3.52 | 1.92 |
|  |  | Positive awe | 4.98 | 1.78 |
| Need for Accommodation | My worldviews changed. | Threat-awe | 4.88 | 1.59 |
|  |  | Positive awe | 4.94 | 1.57 |

S2 Table.

*Zero-order correlations among items of the sense of powerlessness scale*


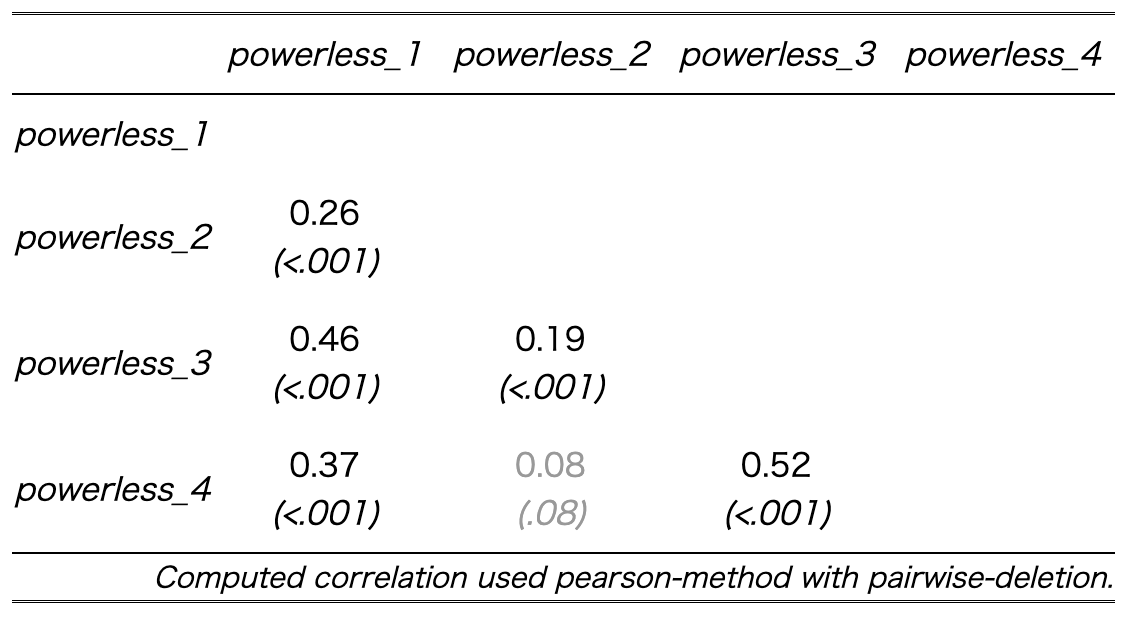


S3 Table.

*Zero-order correlations among items of the interdependent worldviews scale*


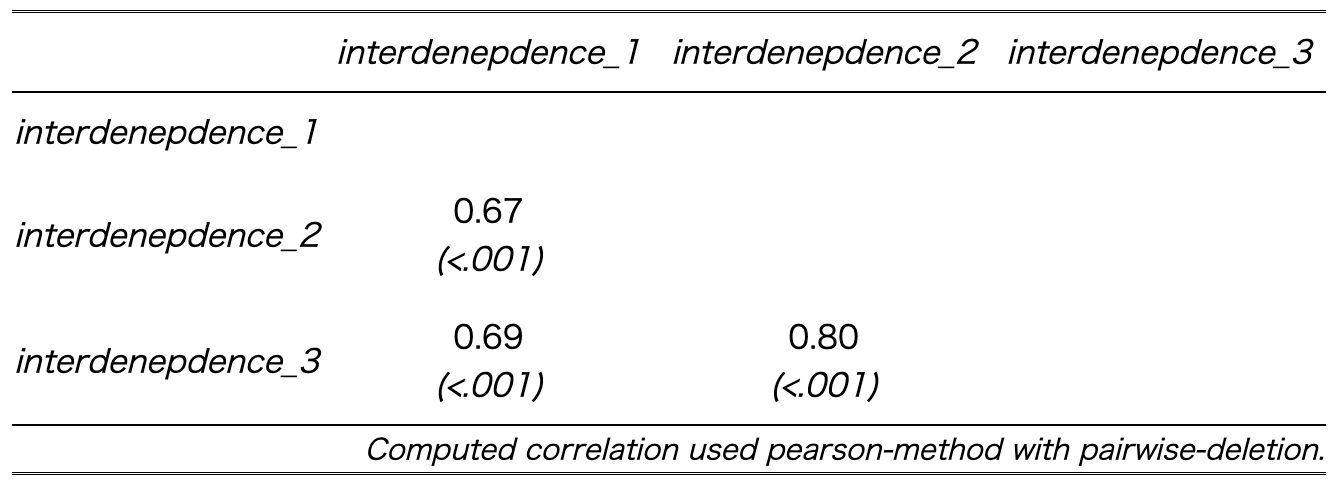


S4 Table.

*Zero-order correlations among main variables (threat-awe condition)*


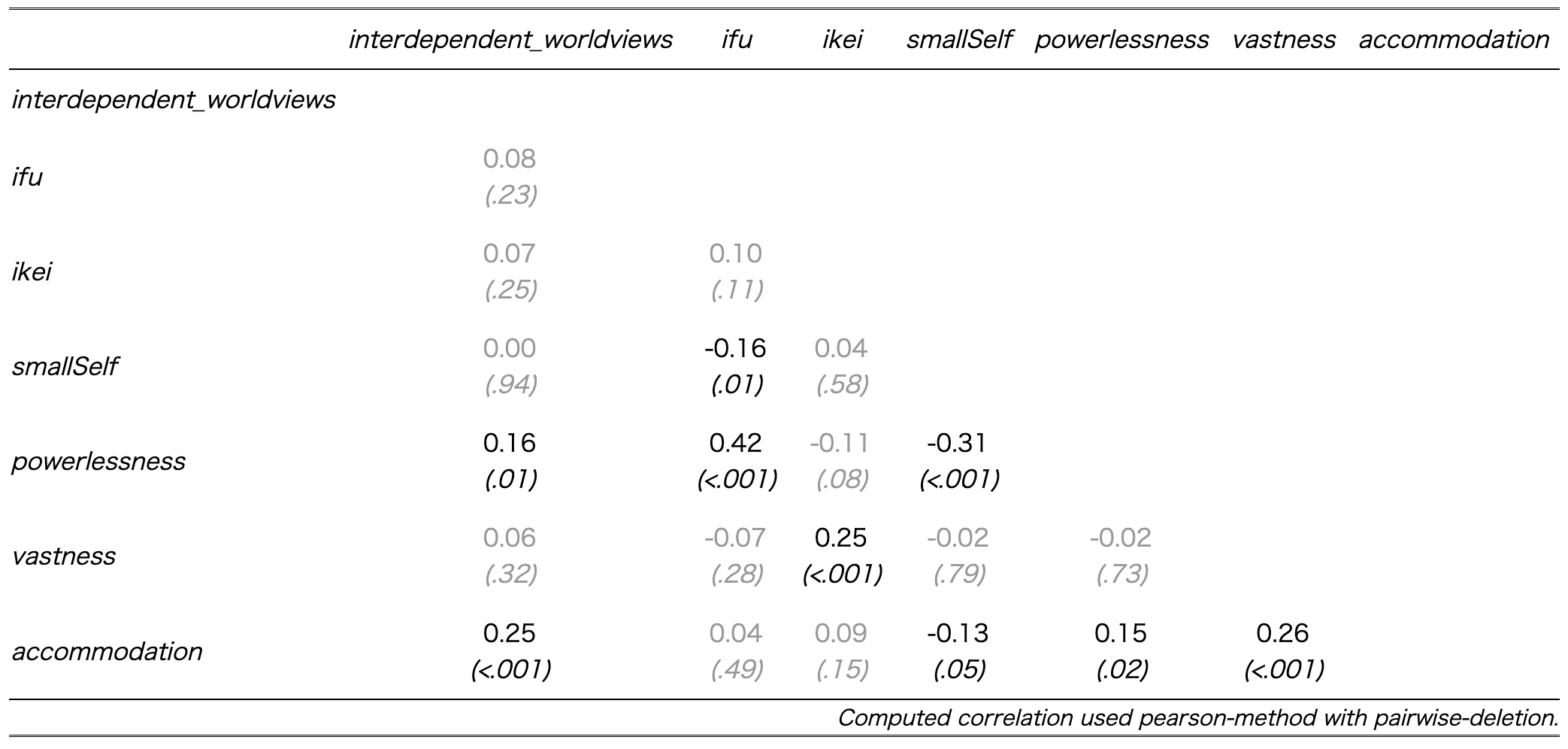


S5 Table.

*Zero-order correlations among main variables (positive awe condition)*


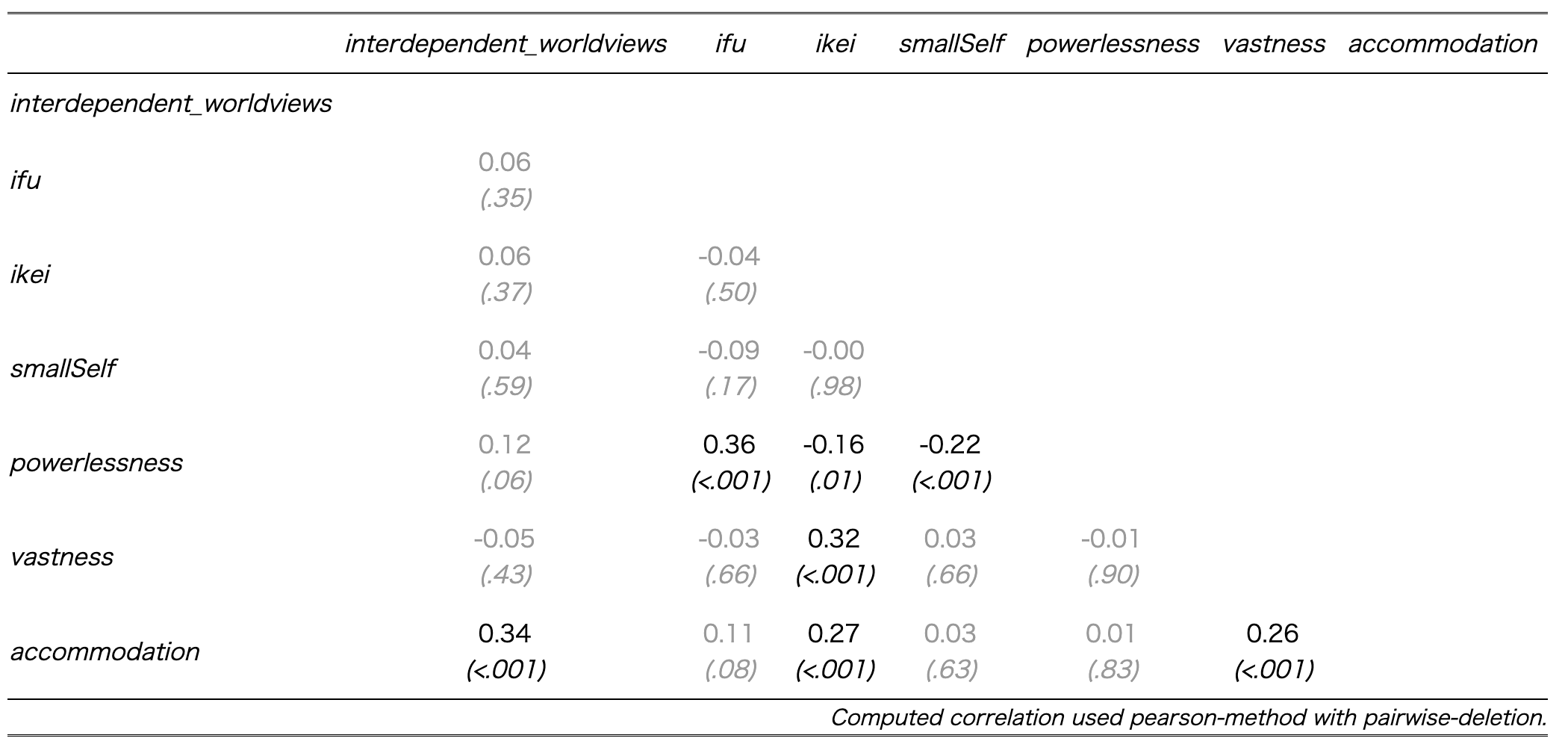


S6 Table.

*Comparisons of threat-awe versus positive awe conditions on variables*

| Variables | Condition | *Mean* | *SD* | *b* | *SE* | *p* |
| --- | --- | --- | --- | --- | --- | --- |
| Powerlessness | Threat-awe | 5.72 | 0.99 | - | - | - |
|  | Positive awe | 5.07 | 1.08 | 0.65 | 0.09 | < .001 |
| Threat | Threat-awe | 6.25 | 1.15 | - | - | - |
|  | Positive awe | 4.93 | 1.93 | 1.32 | 0.14 | < .001 |
| Interdependent Worldviews | Threat-awe | 5.17 | 1.54 | - | - | - |
|  | Positive awe | 4.40 | 1.53 | 0.77 | 0.14 | < .001 |
| *Ikei* (畏敬) | Threat-awe | 5.50 | 2.63 | - | - | - |
|  | Positive awe | 6.81 | 2.18 | -1.31 | 0.22 | < .001 |
| *Ikei* (畏怖) | Threat-awe | 7.66 | 1.60 | - | - | - |
|  | Positive awe | 6.74 | 2.12 | 0.92 | 0.17 | < .001 |
| Small Self | Threat-awe | 1.97 | 1.52 | - | - | - |
|  | Positive awe | 2.03 | 1.48 | -0.06 | 0.14 | .649 |
| Fear | Threat-awe | 7.84 | 1.73 | - | - | - |
|  | Positive awe | 5.34 | 2.87 | 2.50 | 0.21 | < .001 |
| Anxiety | Threat-awe | 7.73 | 1.89 | - | - | - |
|  | Positive awe | 5.22 | 2.87 | 2.52 | 0.22 | < .001 |
| Amusement | Threat-awe | 1.84 | 1.76 | - | - | - |
|  | Positive awe | 4.79 | 2.67 | -2.94 | 0.20 | < .001 |
| Happiness | Threat-awe | 2.22 | 2.17 | - | - | - |
|  | Positive awe | 5.09 | 2.84 | -2.87 | 0.23 | < .001 |
| Gratitude | Threat-awe | 3.02 | 2.57 | - | - | - |
|  | Positive awe | 5.14 | 2.71 | -2.12 | 0.24 | < .001 |
| Wonder | Threat-awe | 6.29 | 2.51 | - | - | - |
|  | Positive awe | 6.64 | 2.24 | -0.35 | 0.22 | .110 |
| Respect | Threat-awe | 2.71 | 2.42 | - | - | - |
|  | Positive awe | 5.56 | 2.75 | -2.85 | 0.23 | < .001 |
| Joy | Threat-awe | 1.96 | 1.89 | - | - | - |
|  | Positive awe | 5.19 | 2.92 | -3.22 | 0.22 | < .001 |
| Sadness | Threat-awe | 6.24 | 2.34 | - | - | - |
|  | Positive awe | 4.08 | 2.74 | 2.16 | 0.23 | < .001 |
| Anger | Threat-awe | 4.16 | 2.54 | - | - | - |
|  | Positive awe | 2.54 | 2.14 | 1.63 | 0.21 | < .001 |
| Coldness | Threat-awe | 5.18 | 2.41 | - | - | - |
|  | Positive awe | 4.24 | 2.55 | 0.94 | 0.22 | < .001 |
| Warmth | Threat-awe | 2.50 | 2.25 | - | - | - |
|  | Positive awe | 4.53 | 2.64 | -2.03 | 0.22 | < .001 |
| Nervousness | Threat-awe | 5.08 | 2.68 | - | - | - |
|  | Positive awe | 3.62 | 2.35 | 1.46 | 0.23 | < .001 |
| Contentment | Threat-awe | 2.00 | 1.89 | - | - | - |
|  | Positive awe | 5.39 | 2.97 | -3.40 | 0.23 | < .001 |
| Vastness | Threat-awe | 3.52 | 1.92 | - | - | - |
|  | Positive awe | 4.98 | 1.78 | -1.46 | 0.17 | < .001 |
| Accommodation | Threat-awe | 4.88 | 1.59 | - | - | - |
|  | Positive awe | 4.94 | 1.57 | -0.06 | 0.14 | .692 |

# References

1. Gordon, A. M., Stellar, J. E., Anderson, C. L., McNeil, G. D., Loew, D., & Keltner, D. The dark side of the sublime: Distinguishing a threat-based variant of awe. J Pers Soc Psychol. 2017;113(2), 310–328. doi:[10.1037/pspp0000120](https://doi.org/10.1037/pspp0000120)
